# Analysis of the energy balance and CO_2_ flow under the influence of the seasonality of climatic elements in a mangrove ecosystem in Eastern Amazon

**DOI:** 10.1007/s00484-021-02224-8

**Published:** 2021-12-01

**Authors:** Antonio Sérgio C. Freire, Maria Isabel Vitorino, Adriano Marlisson L. de Souza, Michell Fontenelle Germano

**Affiliations:** 1 Programa de Pós-Graduação em Ciências Ambientais, Instituto de Geociências, da Universidade Federal do Pará, Rua Augusto Correa, 01, CEP: 66075-110, Caixa postal 479, Belém, PA Brazil; 2 Federal University of Pará (UFPA), Belém, Brazil; 3 Rural Federal University of the Amazon (UFRA), Belém, Brazil; 4 National Institute for Space Research (INPE), São José dos Campos, Brazil

**Keywords:** Carbon, ENSO, Energy, Mangrove, Biometeorology

## Abstract

An unprecedented study was carried out in a mangrove ecosystem in the northeastern coast of the Brazilian Amazon to understand the behavior of climatic elements in a year with the occurrence of El Niño (2015), associated with the seasonal function source/sink of CO_2_ by the ecosystem. Global radiation (*Rg*), net radiation (*Rn*), temperature, relative humidity, precipitation, horizontal wind speed and direction, as well as turbulent flows of sensible heat (*H*), latent heat (*LE*), and carbon (f_CO_2_) were recorded using eddy covariance, a system for studying turbulent flows of heat and gases in the atmosphere. We observed a drastic reduction in rainfall volumes, which accounts for 63.7% of the expected total according to the region’s climatology. Regarding *f*_ CO_2_, the highest values of photosynthesis, autotrophic, and heterotrophic respiration of the ecosystem occurred in the wet season due to precipitation, ideal photosynthetically active radiation, lower soil salinity, and higher NDVI of the ecosystem. In the 2nd semester of the year, we observed that the decrease in cloudiness, causing a higher radiation supply in the forest canopy, accompanied by a reduction in precipitation and an increase in the value of *H* and soil salinity, favored the increase of foliar abscission by the dominant genus *Rhizophora* and *Avicennia*, thus influencing the reduction of magnitudes of carbon source/sink functions in the ecosystem during this season, even on high tide days.

## Introduction

Mangroves are coastal ecological systems typical of estuarine transition areas between terrestrial and marine environments, subject to tidal regimes. They represent a considerable flow of mass and energy, where the balance of this energy and the carbon involved in the biosphere-atmosphere exchange is transformed into organic matter, from simple assimilates in the form of structural and energetic molecules, contributing to the ecological dynamics of different biotic populations, represented by the flora and fauna of this ecosystem (Nagelkerken et al. 2008; Odum et al. 1982; Schedlbauer et al. 2010).

Mangrove forests account for around 8% of the entire coastline of earth’s tropical and subtropical zones, with roughly 137,760 km^2^. Brazil is the third biggest country in mangrove extension, with an area of 9,623.83 km^2^, extending from the State of Amapá to its Southern limit in Santa Catarina. We observe in these ecosystems a significant variation of biological communities, where it operates at certain times of the year as a nursery for a considered variety of invertebrate and vertebrate species (Giri et al. 2011; Lee et al. 2014; Schaeffer-Novelli et al. 2000; Spalding et al., 1997).

Studies on the hourly variability of the main meteorological variables in a mangrove ecosystem in the northeastern state of Pará in the years from 2001 to 2003 verified that the highest average air temperatures occurred in December, while the lowest occurred in April, with the annual average air temperature around 27 °C; relative humidity had an annual average value of 83%. The energy balance showed a significant seasonality demonstrating the local cloudiness’s effect in these energy flows (Silva Junior et al. 2006).

Micrometeorological measurements at mangroves in the Northeastern coast of Pará from November 2002 to August 2003 showed that the seasonal and hourly variations of E and *LE* fluxes and the evaluation of the energy partition and the *Rn* presented high values during the dry season. Bowen’s ratio showed a generally low value in the wet season, indicating a higher proportion of energy was used as latent heat (Pereira and Rodrigues 2013).

Studies on net ecosystem exchange of CO_2_ (NEE), annual net ecosystem production (NEP), and the meteorological and environmental conditions that favor such phenomena in a mangrove forest in Florida have shown that maximum daytime NEE estimated from photosynthetic activity varied from −20 to −25 μ mol (photons) m^−2^ s^−1^ between March and May. The ecosystem’s respiration was highly variable 2.8 ± 2.4 μ mol (CO_2_) m^−2^ s^−1^, reaching its highest values ​​during the summer wet season. During the winter dry season, CO_2_ assimilation in the forest increased due to increased diffuse solar radiation in response to higher radiative transfer in the forest canopy. During 2004, the forest behaved as an atmospheric CO_2_ sink, with net annual ecosystem production around 1.170 g Cm^−2^. This exceptionally high NEP was attributed to year-round productivity and low ecosystem respiration reaching peak values ​​of only 3 g C cm^−2^ d^−1^ (Barr et al. 2010).

Based on the theoretical foundations presented for this ecosystem, it was decided to test the following hypothesis: The ENSO’s influence as a negative anomaly in precipitation over the study area and the consequent modulation of this phenomenon in the differentiated partitioning in the energy balance in the region.

Due to the increase in soil salinity in the 2nd semester of the year and the reduction in rainfall, a fact probably intensified by ENSO, the hypothesis of the reduction of carbon absorption by the forest canopy was also tested due to the occurrence of more significant abscission leaf.

The main goals of this study were to calculate the monthly rainfall accumulation for the year 2015, comparing it with the climatology of the region, analyzing the rainfall anomaly influenced by the South Oscillation Index (SOI), study through the diurnal cycle, considering the Local Time (LT), of *Rg*, *Rn*, as well as the energy balance, through the diurnal cycle of sensible and latent heat fluxes, for the mangrove ecosystem in a year with the occurrence of ENOS. From the observed data, the air temperature and relative humidity were plotted hourly, and the diurnal cycle of the CO_2_ flux (*f*_ CO_2_) was calculated. The behavior of the turbulent fluxes under the effect of the tidal cycles was carefully observed.

## Methodology

### Geological and floristic features of the mangrove in the northeastern coast of the state of Pará

The northeastern region of the state of Pará has, on its coast, a geological formation dating from the Holocene period, where the rise of salinity of the tidal waters in the fluvial sector can be attributed to the sea level rise in the Atlantic Ocean. It is believed that the region may have been subjected to a complex interaction of several factors, mainly consisting of changes in sea level, subsidence rates, and climate changes, the latter being the one with the most significant potential to have affected the discharge of the Amazon river (Behling et al. 2001; Cohen et al. 2012; Pujos et al. 1996; Souza Filho 2005).

Mangroves on the northeastern coast of the state of Pará, known as the *Macrotidal* Amazonian Mangrove Coast, are characterized by a low relief, varying from 0 to 80 m, presenting a vast coastal plain, up to 70 km wide, and an adjacent continental shelf, approximately 200 km wide, being extremely irregular, indented, and cut by several estuaries (Souza Filho 2005).

The floristic features of this ecosystem consist of woody plant species, typical of this biome, with the occurrence of halophytic angiosperms belonging especially to the species *Conocarpus erectus L.*, *Laguncularia racemosa L.*, *Rhizophora mangle L.*, *Rhizophora racemosa* G.F.W. Meyer, *Avicennia germinans*, and *A. schaueriana* (Ferreira 1989; Menezes et al. 2008).

Meteorological observations have shown that in this region, there is the formation of squall lines, sea breeze circulation, and the mean flow is from the Northeast, with an average wind speed velocity of 7 m s^−1^ from 6 to 12 am, LT (Cohen; Silva Dias and Nobre 1995; Germano et al. 2017; Moraes et al. 2005; Silva junior et al. 2006).

Studies on the salinity of the mangrove soil in the municipality of Bragança, Pará have shown that this environmental factor accompanies the rain cycle, when in the wet season, there is a decrease in soil salinity, with a gradual increase in the extent to which there is a reduction of precipitation in the second semester (Koch and Wolff 2002; Mehling, 2006).

### Characterization of the experimental site

**Fig. 1:**
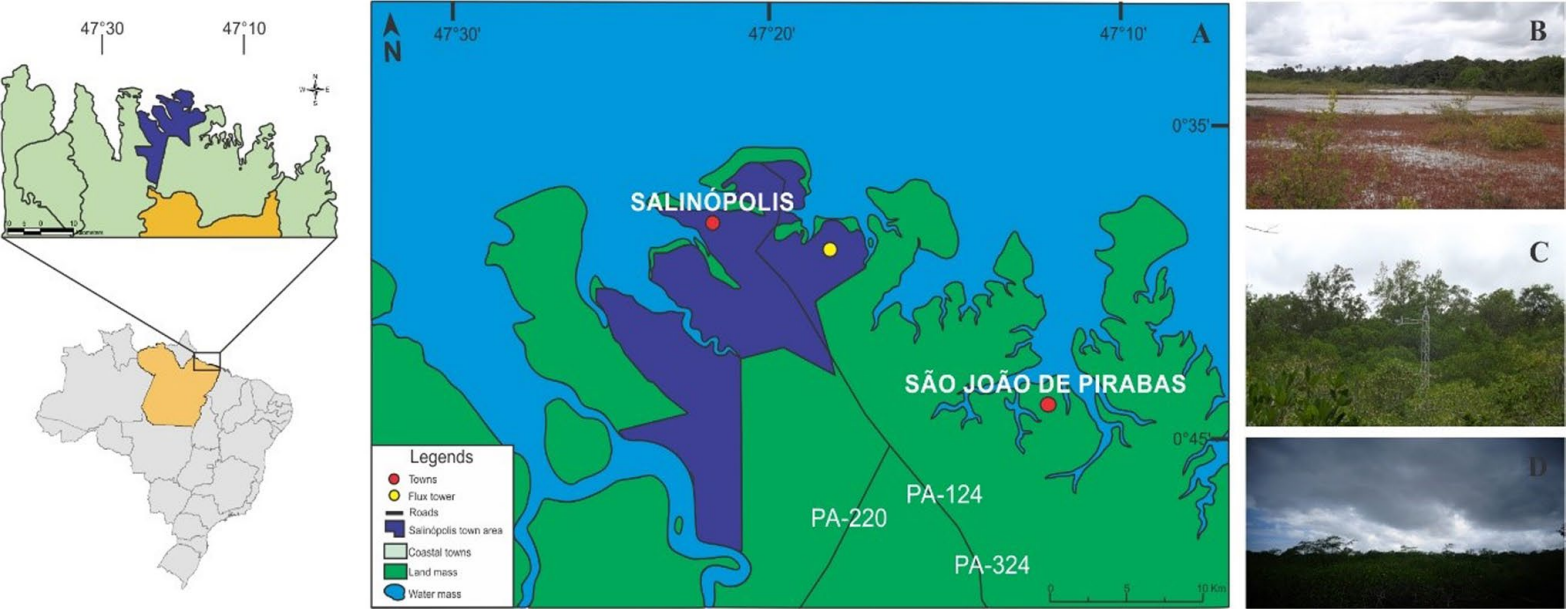
Location of the study area on the northeastern coast of the state of Pará, with emphasis on the municipality of Salinópolis, where the Cuiarana experimental site is located

The micrometeorological data were collected at the experimental site of Cuiarana, a village near the urban center of the city of Salinópolis, Northeastern Pará (Fig. 1A), under coordinates 00 ° 39′50″S, 47 ° 17′10″O. This site presents an area in a state of ecological succession, with typical mangrove tree species, surrounded by mature mangrove (Fig. 1B, D), where the dominance of the genus *Avicennia* (85.56%) and *Rhizophora* (11.47%) occurs in a soil characterized as hypersaline, with trees with average heights of 9.5 $\pm$ 4.5 (m), and the species *Rhizophora mangle L*., *Avicennia germinans* (L.) *Stearn*., and *Laguncularia racemosa* (L.) *Gaertn*., with the following “relative densities” respectively: 30.22%, 58.27%, and 11.51% (De Carvalho and Jardim 2017).

### Instrumentation of the micrometeorological tower

A micrometeorological tower was installed (1C) equipped with eddy covariance (EC), a high-frequency system for studying turbulent flows and low-frequency sensors for the collection of meteorological data.

The mangrove tower’s high and low-frequency sensors were installed in an aluminum structure, which is 10 m high from the ground and 2 m above the forest canopy, providing the “footprint” an equivalent of 1 km^2^ in diameter. The high and low-frequency data integrate two distinct sets of variables recorded in two different data loggers, model CR1000, equipped with a memory card adapter.

The following sensors collected the precipitation data (mm), *Rg* (W m^−2^), *Rn* (W m^−2^), air temperature (°C), and relative humidity (%): rain gauge (TE 525mm), Piranometer-CS 300/Campbell, net radiometer, NR Lite 2, K&Z, the sensor for temperature and relative humidity CS 215/Campbell. The photosynthetically active radiation (PAR) μ mol (photons) m^−2^ s^−1^ was calculated empirically from the *Rg*, according to Aguiar et al. (2012b, a). For the calculation of the radiation budget, we considered Eq. (1) whose terms are expressed by

1 $${\mathrm R}_{\mathrm n}\;=\;\mathrm H\;+\;\mathrm{LE}\;+\;\mathrm G$$

*Rn* is the net radiation, *H* stands for sensible heat, *LE* is the latent heat, and *G* represents the flow of energy propagating in the substrate.

For the energy balance in this mangrove ecosystem, the estimation of soil heat flux (*G*) in Cuiarana was carried out by applying a regression equation between the balance of radiation and the heat flux in the soil measured by Barr et al. (2012), which presented a coefficient of determination with *R*^2^ = 0.63, when they performed similar studies, in the mangrove swamp in Southern Florida, between 2004 and 2009. The reason for applying this methodology was the excellent correlation between the net radiation within wet (0.83) and dry (0.94) seasons among the Everglades mangroves on the Florida coast and Cuiarana on the northeastern coast of Pará state.

In order to obtain the turbulent data, the EC technique was used for the calculation of *f*_ CO_2_, *LE*, and *H*. This system consists of an open-path infrared gas analyzer coupled with a 3D sonic anemometer model CSAT-3A, which measures the three wind direction components in degrees and velocity in ms^−1^, both from Campbell Scientific, Logan, UT, USA. The storage of this high-frequency data (10 Hz) was programmed to generate average values every 30 min, thus totaling 48 continuous data points for every 24 h, considering the LT (Moncrieff et al. 1997).

The EC technique consists of computing the covariance between the fluctuations of the vertical component of the wind velocity *w′*, simultaneously with the scalar of interest’s conservative amount. Therefore, to calculate the carbon flux, we have to compute the covariance of *w′* times the covariance of the concentration of carbon dioxide (Eq. 2); for latent heat flux, we have covariance between *w*′ and specific humidity (Eq. 3); for the *H*, we have to obtain the covariance of *w′* with air temperature (Eq. 4).

2 $$F_c=\rho\overline{w'C'}$$

3 $$LE=\rho\lambda\overline{w'q'}$$

4 $$H=\rho c_p\overline{w'T'}$$

where *ρ* is the absolute density of air; *w'* is the fluctuation of the vertical component of the wind speed; *C′* is the standard deviation from the mean CO_2_ concentration; *λ* is the latent heat of water vaporization; *q′* is the standard deviation from the mean of the specific humidity; C_p_ is the specific heat capacity of the air at constant pressure; *T′* is the standard deviation from the mean air temperature.

Data collection was done monthly, converted into the LoggerNet 4.3 program, and then processed in Alteddy 3.9 software. To calculate the flows of *H*, *LE*, and *f*_ CO_2_, the software was configured to apply a bidimensional rotation to the coordinate system, so the horizontal wind components were aligned with the main flux, and the mean vertical velocity was forced to zero. The Webb correction was used to correct the effects of the humidity of the study area on the temperature measured by the sonic anemometer, in order to adjust the effects of the air density on the measurements of the open path gas analyzer (Webb et al. 1980; Kaimal and Finnigan 1994).

### Method applied to analyze errors in the generated data

The dataset of turbulent fluxes for the year 2015 presented periods with operational failures. These failures were mostly related to wet days, high air humidity, and salinity of the environment, with approximately 93 days without registration and months that were compromised, such as January in which there was no generation of data. For the remaining data processed by Alteddy, quality filters were applied to the output fluxes registers, choosing quality flags from 1 to 3 that point to a better information pattern. Reinforcing this methodological standard, it was also considered the dispose of the fluxes data interval in the hours at which there were night data with u* ≤ 0.20 m s^−1^. Therefore, it was decided not to apply any filling methodology since the filtering of this data was equivalent to 17% of the data generated. After applying these criteria, we obtained 105 and 167 days of reliable data for the wet and dry seasons, respectively.

### Data analysis

#### Precipitation analysis

For comparing purposes of the precipitation variability, an analysis of monthly accumulated data for 2015 was carried out. These data were compared with the 33-year climatology (1978 to 2010) of the Brazilian National Water Agency (ANA) meteorological stations in Salinas’ municipality. Considering the precipitation of 2015, we carried out a study for the anomalies associated with the analysis of the Southern Oscillation Index (SOI), obtained from the NCEP/NOAA website, in order to understand the effects of the sea surface temperature (SST) of the Pacific Ocean, under the precipitation in the Amazon.

#### Energy analysis

For the data of *H*, *LE*, and *f*_ CO_2_, retrieved from the EC system, as well as *Rg* and *Rn*, we calculated the diurnal cycle to understand how these variables behave hourly in a year (2015) of El Niño with a magnitude of 1.25. The PAR was used to understand the hour and spectral range that allows the efficiency of photosynthesis.

*Rg* and *Rn*’s data and *H* and *LE* obtained by the EC technique were transformed into hourly means to generate 24 data points, so there would be a representation for every hour of the day for 2015.

#### Soil salinity data and leaf area of the mangrove canopy

Two analyses were carried out to test the hypothesis of the seasonal behavior in carbon fluxes from the foliar abscission. The first one being on the salinity of the water of the soil pores in an area of 1 km^2^ on the periphery of the EC tower with a handheld refractometer ATAGO, where six samples were collected in April 2015 with an average result equal to 32.3 ppt and another six samples collected in November 2015, with an average result of 70 ppt.

The second analysis was through remote sensing using the Normalized Difference Vegetation Index (NDVI) from Landsat 8 channels 3 and 4 (red and infrared respectively) for the months that showed the best condition analysis. Therefore, the images were selected for February and September of 2015 to justify foliar abscission by chlorophyll’s radiation’s reflectance. In February, we verified an NDVI of 0.75, and for September, the NDVI was 0.69 $\pm$ 0.04.

#### Tidal cycle

Another data analyzed was the local tidal cycle, classified as semidiurnal and weakly asymmetrical, with a flood period of up to 6:40, with records of maximum heights of 5.5 m during the spring equinoxes. Currents have a predominant northwest direction during flood tides and southeast direction at reflux tides. The highest current velocities were recorded during the flood tide, with a maximum of 0.5 m/s in March and September, while for the backflow tides (March and June), the maximum velocities reach a value of 0.4 m/s (Pinto et al. 2011).

The tide cycle information for the study year was retrieved from the naval anchorage in Salinópolis, semidiurnal. Only the highest tides’ information was considered since they presented soil flood possibilities for the Cuiarana mangrove.

#### Computation of seasonal energy flow and balance under tidal cycle effect

To analyze the measurements of the turbulent energy fluxes (*H*, *LE*, and *G*), under the influence of high and dry tides during wet and dry seasons, the months of April and October were selected to represent both seasons, respectively. The four highest irradiance times (11:00 am, 12:00, 1:00 pm, and 2:00 pm) were selected, constituting the four data points analyzed in the days of the months studied under the tidal cycle’s influence.

In April, the Julian days from 99 to 102 represented the flows during the high tide, and the days from 105 to 108 represented the days for the low tide. For October, the Julian days from 277 to 280 represented the days analyzed for the high tide, while from 297 to 300, represented the days of low tide. The selected data were averaged for the selected days’ interval, generating a single value for each variable for April and October under high/low tide influence.

## Results and discussions

### An analysis of meteorological data for 2015: air temperature, relative humidity, and precipitation, with its respective anomaly

The daily analysis of the air temperature in the mangrove ecosystem showed that March had inverse values for the relative humidity throughout the year, which we can highlight the biggest temperature occurring on December 11 with 31.2 °C at midnight, while the lowest temperature occurred on March 7 with 23.9 °C at 5:30 am. The diurnal cycle of the air temperature for 2015 shows that between 10 am and 4 pm, this variable reaches and maintains values between 27 and 28 °C for almost 6h.

The relative humidity maintained its highest values during the wet season with gradual decay during the wet season transition. In the diurnal cycle, the highest air humidity values were 77.8% and occurred at 9:00 am; the lowest values were recorded at 5:00 pm, with 64.7%.

The accumulated monthly precipitation data for 2015 showed a total of 1.73 mm. When compared to the climatological normal of 33 years (1978 to 2010) for the municipality of Salinópolis, whose accumulated annual value was 2.72 mm, we verified that the precipitation in 2015 accounted for 63.7% of the total expected, with a deficit of 988 mm. Regarding the rainfall volume of 2014, which had an annual accumulation of 2.60 mm, we verified that in 2015 it rained only 66.6% of the previous year’s volume, representing a deficit of 868.6 mm compared with 2014.

**Fig. 2:**
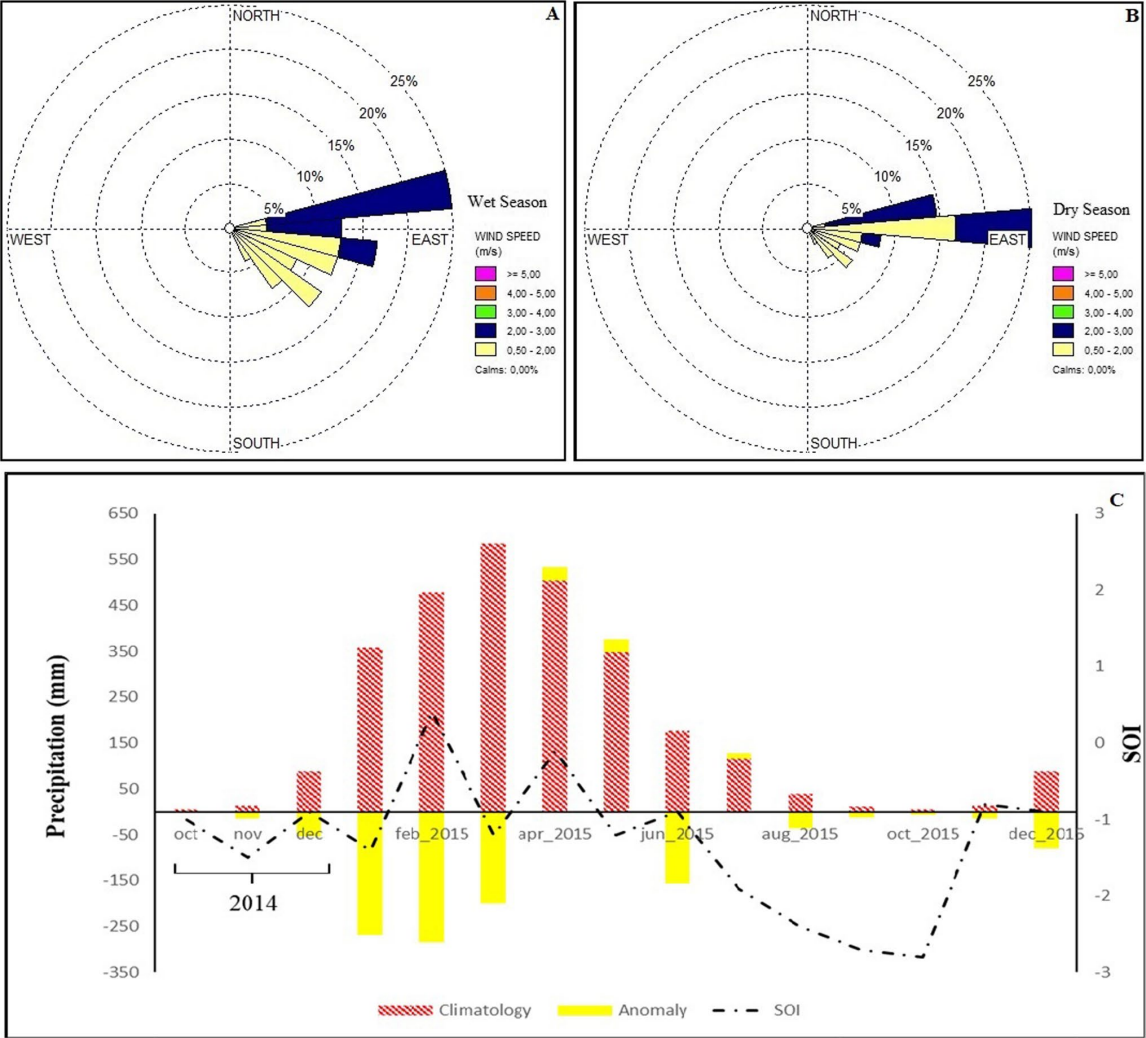
Thirty-three-year climatology (1978 to 2010), accompanied by the calculation of the monthly anomaly for the year 2015 under the influence of the Southern Oscillation Index (SOI) (**C**) and characterization of the wind speed and direction for each season: wet season (**A**) and dry season (**B**)

Data from the NCEP/NOAA (2017) showed the Brazilian Amazon under the influence of the El Niño Southern Oscillation (ENSO) in 2015, whose annual average magnitude of the El Niño oceanic index was 1.25 with greater amplitude in the fourth quarter of 2014 (October, November, and December) (2.3), thus influencing a considerable reduction in precipitation seen in the region in 2015 (Fig. 2C).

During the wet season (from January to May 2015), the total precipitation accumulated was 1.58 mm, representing 69% of the total expected compared to this season (2.27 mm). The early months of the wet season (January to March) presented negative precipitation anomalies (696.1 mm). It must be observed, with particular attention, that these months are known for high precipitation volumes.

According to climatic studies by Pereira and Rodrigues (2013) and Silva Junior et al. (2006), in the mangrove ecosystem of Bragança (State of Pará), the months of February, March, and April are the rainiest, with March being the one with the highest accumulated precipitation, with values above 700 mm for the years from 2001 to 2003.

During the dry season of 2015 (August to December), the observed rainfall was quite anomalous compared to the season’s total expected. The total precipitation accumulated for the season was 12.3 mm, whereas the climatology expected 159.8 mm. Therefore, in these months, it rained only 7.7% of the total expected for the season.

Silva Junior et al. (2006) reported a similar precipitation behavior to what we have seen in Cuiarana, with a reduction in precipitation in the dry season, noting that from August to November, rainfall accounted for 0.2% of the total annual volume for the years from 2001 to 2003.

Zeng (1998), when conducting studies on the seasonal cycle and interannual variability of the Amazonian hydrological cycle, found that there is a good positive correlation between the SOI and precipitation anomalies occurring in the Amazon, with a temporal lag of 3 to 4 months related to events occurring in the Pacific equatorial. Therefore, it should be noted that the SOI of −1.5 in November 2014 was the largest negative anomaly of this year. It may have influenced the anomalous amounts of precipitation seen in February and March 2015 (Fig. 2).

Marengo (1992) and Marengo and Nobre (2009) observed that the subtropical jet in the upper troposphere during the austral winter is more intense and closer to the equator than in the summer, being associated with the decrease of the convection in the Amazon Rainforest. Both authors verified that during the austral winter, the circulation in the low troposphere is characterized by the northernmost position of the Equatorial Trough in the region, decreasing the trade winds’ intensity and the moisture advection from the Atlantic Ocean.

These observations reinforce the discussion of Nobre et al. (2009). They state that local tropical convection constitutes one of the main processes for the formation of precipitation in the entire Amazon basin, being modulated by large-scale circulations, such as the Hadley cell, the positioning of the Intertropical Convergence Zone (ITCZ), and the zonal circulation of the Walker cell.

De Souza et al. (2004), Marengo and Hastenrath (1993), and Ronchail et al. (2002) found that the weakening of convection in the Amazon Basin during El Niño events is explained by a change in the induced subsidence by the Walker circulation. These authors show that the zonal displacement of the Walker cell, as a consequence of the ENSO, favors the reduction of the upward movement during El Niño events over the north and northeast sectors of South America, which triggers the reduction of the precipitation in the Amazon.

We suspect that the occurrence of SOI for July (−1.9), August (−2.4), and September (−2.7) of 2015 influenced the low rainfall in September (0.0 mm), October (0.0 mm), and November (0.1 mm) of this year, with a great reduction in rainfall for the region when the climatology for this quarter expected a volume of 31.7 mm.

### Analysis of the breeze circulation and the mean flow

The observations for wind direction and speed at the Cuiarana (Fig. 2A, B) experimental site agree with Germano et al. (2017) studies on the breeze circulation in Eastern Amazon. The diurnal cycle of the wind direction shows a maximum of northeast and east, with an average wind speed of 2.21 ms^−1^, for the season between 10:00 am and 5:00 pm, during the wet season and 3.0 ms^−1^ in the dry season, characterizing the occurrence of the sea breeze (SB). The SB occurs in the same direction as the trade winds (east), being more frequent from 9:00 am to 6:00 pm LT.

We observed that the LB occurred in the direction of southeast/south (Fig. 2A), during the wet season, with a time of occurrence between 00:00, and 6:00 am LT. However, we should notice that the LB circulation is less intense than SB since it opposes the mean flow direction. The LB was weaker for the dry season than the wet season; the average wind speed for this circulation was 1.0 m s^−1^.

We also observed that the horizontal wind speed increases during the day (Fig. 2B), with the occurrence of thermal turbulence associated to the SB, and also because this flow is located in the same quadrant of the trade winds, thus increasing the wind speed due to the superposition of the SB with the mean flow. We verified that increasing the horizontal wind speed from 1.0 ms^−1^ (Fig. 2B) promotes a decrease in CO_2_ concentration of 500 parts per million/volume (ppmv) to 350 ppmv; these results concur with the findings by Silva Junior et al. (2004) for diurnal CO_2_ concentration in a pasture area in the Amazon forest.

### Studies of global, liquid radiation, and energy fluxes for the wet and dry seasons

The general observations of the radiation balance data on the mangrove ecosystem showed that the highest magnitudes for *Rg*, *Rn*, and *H* occurred in the dry season, while the values for the *LE* were higher in the wet season.

**Fig. 3:**
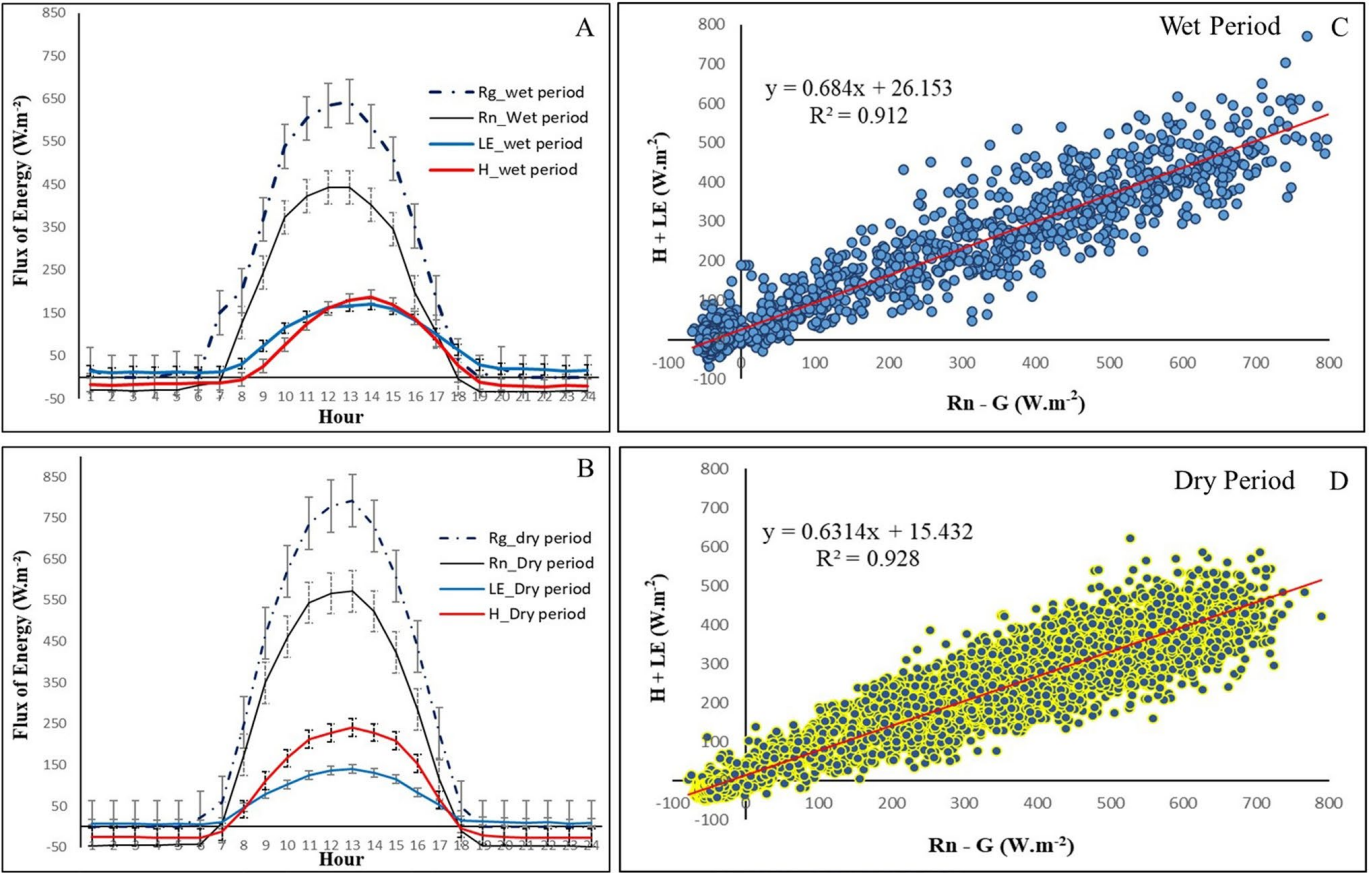
Day cycle of *Rg*, *Rn*, *LE*, and *H* during the wet (**A**) and dry season (**B**) for 2015. The relation between the available energy in the system (*Rn*−*G*) and the sum of the sensible and latent heat fluxes (*H* + *LE*) wet (**C**) and dry season (**D**); associated with Table 1, with mean values (with standard error) for hours with the highest irradiance (11:00 am to 2:00 pm) for the sensible (*H*), latent (*LE*), net radiation irradiance (*Rn*/*K*_in_), *H*/*Rn*, the evaporative fraction (*LE*/*Rn*), soil heat flux (*G*), and the energy balance closure (*H*+*LE*)/(*Rn*−*G*), for the wet and dry season of 2015 at the experimental site of Cuiarana

*Rg*’s data show that at 1:00 pm, we observed the highest magnitudes of the net shortwave radiation, with 673.8 W m^−2^ $\pm$ 24.15 W m^−2^ in the wet season and 792.2 $\pm$ 10.7 W m^−2^ in the dry season (Fig. 3A, B). For the *Rn*, we observed at 1:00 pm, for both wet and dry seasons, the highest magnitudes, with 469 $\pm$ 18.2 W m^−2^ and 572.2 $\pm$ 7.9 W m^−2^, respectively.

When we have the maximum shortwave and diffuse radiation (2:00 pm) from the dry to the wet season, the radiative difference is 148.4 W m^−2^. In comparison, the net radiation at 1:00 pm is 103.9 W m^−2^, confirming that the seasonality in the region is very pronounced, characterized by the decrease of cloudiness, due to the displacement of the ITCZ, in the months of the dry season, allowing a more significant entrance of radiation to the surface, favoring greater energy availability for the physical processes in the lower troposphere. Fernandes et al. (2018), studying the climatic configuration through the radiation balance and transmissivity indexes (*Kt*) of shortwave radiation in the mangrove ecosystem in Cuiarana, verified that for February, March, and April, the *Kt* assumes the lowest values of the year due to the higher reflectance of the radiation in this season, with a consequent decrease of the energy availability in the forest canopy.

The analysis of energy partition between latent heat and sensible heat for the two seasons shows that the *LE* predominates in energy processes much longer throughout the day (at night and dawn) and that the magnitude of *H* is more significant than *LE* in the wet season for 5 h, between 11:00 am and 4:00 pm, where the maximum values for *H* and *LE* occurred at 2:00 pm with 187.7 W m^−2^ $\pm$ 3.8 and 171.2 W m^−2^ $\pm$ 4, respectively. However, this observed feature in the wet season is marked by a very close partition between *H* and *LE* during the day, ratifying the water availability in the system in the form of moisture, rainfall, and tidal pulses, making possible the efficiency of the physiological phenomena of the mangrove, mainly in the gas exchanges between water vapor and atmospheric CO_2_, to increase biomass through photosynthesis (Fig. 3C).

From June to December, between 9:00 am and 5:00 pm, the energy partition reveals that the magnitude of *H* in relation to the *LE* prevails for 8 h in this season, with a maximum value at 1:00 pm with 241 W m^−2^ $\pm$ 4.1. This environmental behavior of the ecosystem is expected due to the decrease in cloud cover, with a consequent reduction in rainfall volumes, as well as the decrease of air humidity in the second half of the year, where the highest *LE* value was also observed at 1:00 pm with 139.5 W m^−2^ $\pm$ 3.4 (Fig. 3B).

Pereira and Rodrigues (2013), analyzing the energy partition in the mangrove ecosystem on the coast of Pará, found that in the wet season, the maximum value of *H* occurred in January at 1:00 pm with 274.7 W m^−2^. On the other hand, the maximum *LE* observed occurred in May at 11:00 am with 374.7 W m^−2^. During the dry season, *H*’s maximum value occurred in November at 11:30 am with 300.2 W m^−2^; the *LE* had its maximum value in June at noon with 267.5 W m^−2^ (Fig. 3B).

We should notice that the data recorded by these authors with their maximum values higher than those observed in Cuiarana and differences in the hours occurred due to the location of the meteorological tower. The meteorological tower in Bragança was installed in a deforested area, thus justifying a higher reflectance of the soil with few vegetal cover.

Studies of Barr et al. (2014) about seasonal mangrove evapotranspiration revealed that diurnal and seasonal controls of water vapor fluxes evidenced that the energy partition between *H* and *LE* was highly variable. The forest behaved as a semi-arid ecosystem during the dry season, with most energy partitions converted into *H*, and minimum *LE* values with 5 MJ d^−1^. In contrast, during the wet season, the forest presented *LE* fluxes with 18 MJ d^−1^.

The dry season, high salinity levels were observed, influencing the reduction of evapotranspiration and reducing the stomatal canopy conductance. These authors verified that the canopy’s daily conductance to the water vapor decreased with increased salinity from the multiple linear regression analysis.

### Study of incident radiation (K_in_), fluxes of H, LE, and G and energy balance

Data of the incident radiation (*K*_in_) represented by the *Rn*/*K*_in_ relation for the days and hours studied showed that both *K*_in_ and the net radiation in the ecosystem presented the seasonal and intraseasonal patterns for eastern Amazonia. This energy availability governs the physical and biological processes in the mangrove and the behavior of the other elements of the energy balance (Table 1).

**Table 1:** Mean values (with standard error) for the hours with highest irradiance (11:00 am to 2:00 pm)

Month	*H* (W m^−2^)	*LE* (Wm^−2^)	*Rn*/*K*_in_	*H*/*Rn*	*LE*/*Rn*	*G* (Wm^−2^)	*H*+*LE*/*Rn*−*G*
Wet season							
Mar	169.1 ± 1.9	175.2 ± 1.9	0.94	0.35	0.36	11.5 ± 0.3	0.7
April	172.8 ± 2.2	212.6 ± 2.5	0.93	0.32	0.39	11.8 ± 0.1	0.7
May	144.8 ± 1.8	183.6 ± 0.3	0.72	0.30	0.38	11.6 ± 0.3	0.7
June	175.4 ± 2.3	189.1 ± 1.7	0.77	0.31	0.34	9.8 ± 0.2	0.6
Dry season							
Aug	230.9 ± 2.9	139.4 ± 1.1	0.74	0.40	0.24	19.2 ± 0.1	0.6
Sep	261.3 + 2.7	140.3 ± 2.2	0.72	0.43	0.23	21.3 ± 0.1	0.7
Oct	228.6 ± 1.8	111.5 ± 0.7	0.71	0.42	0.20	20.2 ± 0.2	0.6
Nov	235.7 ± 2.1	113.2 ± 1.7	0.71	0.44	0.21	18.7 ± 0.1	0.7

The energy partition analysis in the Cuiarana mangrove showed that the energy balance closure through the ratio (*H* + *LE*)/(*Rn*−*G*) had a deficit of ~ 30% in the wet ~ 40% in the dry season. We should notice a predominance of *LE* concerning *H* during the months of the wet season. The main reasons for this are the precipitation of the first half of the year, the high humidity of the air, and tidal pulses, thus allowing a more significant amount of water vapor to the soil-biosphere-atmosphere system.

The evaporative fraction (*LE*/*Rn*) presented better results in the wet season months, with values similar to (*H*/*Rn*). In the second half of the year (dry season), we observed a decrease in the *LE* /*Rn*, with mean values around 0.20 and *H*/*Rn* elevation for mean values around 0.40 (Table 1).

We noticed the dry season data, the seasonality’s characteristic effects, with a predominance of *H* compared to *LE*, with an average value of 239 W m^−2^ for *H* between the months in the second half of the year. The effect of seasonality on energy partition is due to the region’s hydrological and energetic cycle’s different behavior, reflecting the decrease in *LE*/*Rn* values to ~ 20% and *H*/*Rn* elevation ~ 40% (Table 1).

The soil heat flux (*G*) accompanies the seasonality of energy availability in the ecosystem, with mean values of 11.1 W m^−2^ between March and June and 19.5 W m^−2^ between August and November. The lowest/highest energy availability in the wet/dry seasons reflects the region’s large-scale weather systems (Table 1).

The energy balance closure presented showed a slope of 0.68 and a determination coefficient of 0.91 for the wet season. For the dry season, the line’s slope was 0.63, and the determination coefficient showed values of 0.92. The slope found is consistent with the values found in studies on mangrove ecosystems that used the EC technique, citing 0.82 in Barr et al. (2013).

### Seasonal study of the effect of the tidal cycle on the mangrove energy balance

The energy partition during the wet and dry seasons, under the influence of the tidal cycle, showed that there was a predominance of *H* concerning the *LE* during the occurrence of low tides in both seasons (*H*= 203.3 ± 3.1 W m^−2^; *LE*=193.3 W m^−2^/0.25m and *H*= 241.5 ± 2.7 W m^−2^; *LE*= 119.4 W m^−2^/0.3m) and predominance of *LE* concerning *H* only during high tides (*LE*= 202.1±2.2 W/m^−2^; *H*= 172.1 W m^−2^/ 4.3 m) from the wet season. In the dry season, the *LE* was lower than *H* (*LE*= 125±1.7 W m^−2^; *H*= 232 W m^−2^/4.1 m) (Table 2).

**Table 2:** Average energy fluxes for 4 days in April (high/low tide) and 4 days in October (high/low tides) at the highest irradiance times (11:00 am to 2:00 pm), with the respective energy partition and standard errors

	Wet season		Dry season	
	April		October	
	High tide	Low tide	High tide	Low tide
Wind speed (m/s)	2.2	2.4	3.0	3.0
Tide height (m)	4.3 ± 0.02	0.25 ± 0.1	4.0 ± 0.1	0.3 ± 0.1
*H* (W m^−2^)	172.1 ± 4.6	203.3 ± 3.1	232.8 ± 4.2	241.5 ± 2.7
*LE* (W m^−2^)	202.1 ± 2.2	193.3 ± 2.8	125 ± 1.7	119.4 ± 0.7
*G* (W m^−2^)	15.16 ± 0.6	19.7 ± 0.3	11.5 ± 0.1	11.6 ± 0.1
*H*/*Rn*	0.34	0.38	0.41	0.43
*LE*/*Rn*	0.40	0.36	0.22	0.21
*G*/*Rn*	0.03	0.04	0.02	0.02
(*H*+*LE*)/(*Rn*−*G*)	0.74	0.73	0.64	0.64

We verified that the tidal cycle in the region influenced the energy partition, with the region’s seasonal characteristics. In the first half of the year, we noticed that the high cloud cover in the local atmosphere, coinciding with the high tides, favored the predominance of *LE*, while in the second half of the year, with lower rainfall availability and higher incident radiation supply, there was a consequent predominance of *H* concerning other energy flows, both in the high and low tides.

Regarding the soil heat flux (*G*), we observed that during the wet season’s low tides, the highest magnitudes of energy occurred with 19.7 W m^−2^, equivalent to 4.7% of the *Rn*. During the dry season’s low tides, the average *G* fluxes were around 9 W m^−2^, which corresponds to 3% of the *Rn*.

The analysis of the energy balance closure for the high/low tide hours in both seasons showed that the better energy balance closure occurred during the high tide of April, with 0.74, evidencing that the energy transfer associated with tidal activity improved the energy balance of the flooded surface during the observation season, even though the wind speed was 2.7 m s^−1^.

Analyses by Barr et al. (2013), which quantified the energy transport, during the high tides (and low tides) in a mangrove in Southern Florida, by summing the enthalpy change (Δ*H*_tot_ = total enthalpy variation (Δ*H*_tot_ = Δ*H*_stor +_ Δ*H*_adv_)) of heat stored (or released) in the water column (Δ*H*_stor_) and the heat exchange advected by the tidewater (Δ*H*_adv_), verified that when Δ*H*_tot_ was included in the energy balance, at the times of the day with the greatest irradiance, the energy balance closure improved from 0.73 to 0.75. Therefore, these authors verified that the tidal waters attribute an energetic computation improving the microclimatic conditions within the forest canopy through these observations.

Regarding the source/sink function of CO_2_, under tidal effects, we noticed that when there was a supply of water vapor in the low troposphere, during the high tides, especially during the wet season, carbon assimilation was accentuated (12.3 μ mol (CO_2_) m^−2^ s^−1^) by the stomatal exchange in the forest canopy. During the low tide in April, the average carbon assimilation was 16% smaller (10.3 μ mol (CO_2_) m^−2^ s^−1^). In October, carbon assimilation was 9.3 μ mol (CO_2_) m^−2^ s^−1^ on the days of high tide, while during the low tides, the forest absorbed 9.0 μ mol (CO_2_) m^−2^ s^−1^, corroborating the fundamental role of water in the stomatal exchange and improvement of carbon assimilation, mainly within the local seasonal characteristics.

### Study of the diurnal carbon cycle

The CO_2_ diurnal cycle during 2015 under the effects of ENSO showed that carbon sequestration in both the wet and dry season begins around 7:00 am when the solar zenith angle initiates the process of photosynthesis in the forst. A reduction of the mangrove’s photosynthetic physiological activity occurs around 5:30 pm in the wet season and 6:00 pm in the dry season (4A).

**Fig. 4:**
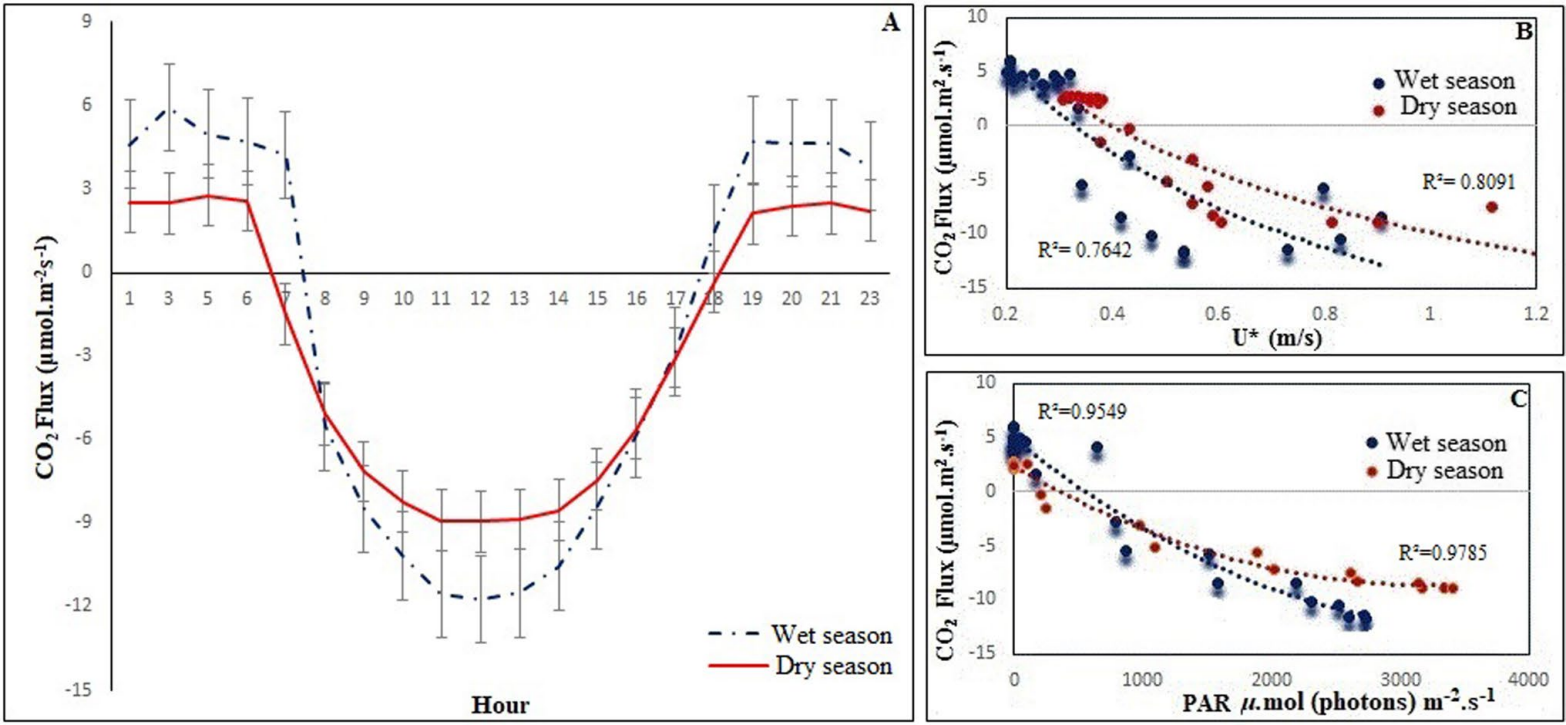
Diurnal cycle of the carbon flux for the months in the wet and dry season in 2015 at the Cuiarana experimental site, Pará

From 6:00 pm, the ecosystem is performing only autotrophic and heterotrophic breathing. We should notice that along the year in different hours when PAR occurred between 1.9 μ mol (photons) m^−2^ s^−1^ and 3.1 μ mol (photons) m^−2^ s^−1^, there was a predominance of photosynthesis concerning the respiration of the ecosystem (Fig. 4C).

We observed that photosynthesis was predominant in the wet season compared to the dry season. The precipitation in the first half of 2015 was associated with lowering horizontal wind speed, and a decrease in soil salinity, which favored the maximum photosynthetic activity, between noon with −12.9 μ mol (CO_2_) m^−2^ s^−1^ and 1:00 pm −13.5 μ mol (CO_2_) m^−2^ s^−1^ (Fig. 4C), where the PAR showed maximums of 2.727 μ mol (photons) m^−2^ s^−1^ in the wet season and 3.406 μ mol (photons) m^−2^ s^−1^ in the dry season. These results agree with observations made by Barr et al. (2014) and Leopold et al. (2016), associated with the mangrove phenology in delaying foliar abscission, as described by Nascimento et al. (2006).

Barr et al. (2010), studying the net CO_2_ exchange between the mangrove ecosystem and the coastal atmosphere of FL, USA using the EC technique, found that maximum daytime CO_2_ absorption ranged from −20 to −25 μ mol (CO_2_) m^−2^ s^−1^ between March and May (Spring). During the winter, the mangrove’s CO_2_ assimilation increased by the same proportion as the diffuse solar radiation due to the radiative transfer in the forest canopy.

The analysis of the data of the respiration of the ecosystem showed that the magnitudes of this physiological phenomenon were higher in the wet season, with peaks of 10 μ mol (CO_2_) m^−2^ s^−1^ at 4:00 am, while in the dry season, we registered 2.8 (CO_2_) m^−2^ s^−1^ at 5:00 am. The physiology of respiration was expected to have higher magnitudes in the second half of the year, due to a decrease in cloudiness and consequent increase in radiation availability, as observed by Barr et al. (2010).

However, we do not observe this in the ecosystem on the coast of Pará, by the seasonality of the region marked by the reduction of precipitation and elevation of salinity of the water of the mangrove soil from 32.3 ppt in the wet season and 70 ppt in the dry season, allowing the species *Avicennia germinans* and *Rhizophora mangle* to show more significant adaptation and dominance in this hyper hyaline ecosystem, as shown by studies of Menezes et al. (2008), Laurance et al., (2004), Do Nascimento et al. (2006), and ratified by Fernandes (2016).

Studies by Barr et al. (2014) on soil water salinity’s seasonality to support seasonal mangrove evapotranspiration in Southern California found that the salinity varied the dry season rapidly at 35 ppt, to values between 26 and 17 ppt at the beginning of the wet season. The increase of precipitation in June and July and the increase of river discharges contributed significantly to the salt dilution of the soil’s micro and macropores.

Observations by Leopold et al. (2016), in the “Coeur de Voh” mangrove in New Caledonia, also verified that the salinity of the water presented a high seasonal variation. The salinity of water from the soil interstice was much lower during the wet season than the dry season. These authors verified that the decrease of salinity was related to the system’s freshwater intake during the wet season. It was also observed that the high volumes of precipitation increased the discharge of the river and consequent contribution to the tides flood with high freshwater content, which may have diluted the concentration of the saline solution from the soil interstices.

Seasonal remote sensing data for the Cuiarana mangrove for 2015 showed a variation of the vegetation cover through the NDVI analysis of the forest canopy between the wet and dry season values of 0.75 $\pm$ 0.05 and 0.69 $\pm$ 0.04, respectively. We noticed a variation in the forest canopy from the chlorophyll infrared reflectance, suggesting a decrease in the leaf area index.

Therefore, with the beginning of the dry season, the genus *Avicennia* and *Rhizophora* assume, as an environmental adaptation strategy, the foliar abscission, reducing water vapor by leaf area, and acquiring the lowest values source/sink function of carbon in this season.

Cunha et al. (2006); Fernandes et al. (2007); Menezes et al. (2008) attribute to this phenological behavior of *Avicennia* and *Rhizophora* the striking feature of salinity seasonality and tidal levels. However, observations by Cunha et al. (2006), studying the frequency and structure of three mangrove species in Southeastern Brazil, and the observations through the EC technique for Cuiarana, showed that other meteorological elements with a synoptic scale influence the adaptive behavior of these genus in the mangrove, such as the meridional shift of ITCZ to its northernmost position, reducing the cloudiness in the region, with consequent reduction of precipitation, thus influencing the energy partition and *LE* reduction for the second half of the year.

In this way, with less water in the system, to make possible the stomatal changes in the most demanding season, with high air temperature, reduction of the relative humidity, and higher incident radiation, the species, in order to avoid water stress, promote foliar abscission in this season, corroborating the observations of Fernandes et al. (2007), in the Bragança mangrove that described the seasonality of foliar abscission of the genus *Avicennia* and *Rhizophora* within a same seasonal pattern that has been observed in Cuiarana through the CO_2_ flux.

## Conclusions

For 2015, under ENSO in the mangrove ecosystem of the experimental site of Cuiarana, on the northeastern coast of the state of Pará, we verified that the precipitation had volumes below the climatological normal, evidencing the strong influence of the sea surface temperature anomalies in the Pacific Ocean over the Amazon. Therefore, the energy partition was affected by this precipitation anomaly, inducing a predominance of *LE* at various times of the day, influenced by the small precipitation volumes. In the dry season, the *H*, temperature, and relative humidity followed the climatological normal expected for the region.

Regarding the carbon sink/source function performed by the mangrove forest, where there is a great abundance of the species *Avicennia germinans* and *Rhizophora mangle*, we verified that the largest magnitudes of photosynthesis and respiration occurred significantly during the wet season, since a series of physical factors such as lower soil salinity, high precipitation, cloudiness attenuating the radiation, and biotic factors such as transpiration through stomatal activity, favored the physiology of the ecosystem. However, there is an inversion of these physical patterns in the dry season, accompanied by foliar abscission and decrease in the mangrove tree species’ physiological functions.

The tidal cycle effect was analyzed both in the partition of energy and in the source/sink function of carbon. We observed a favorable condition for carbon absorption on high tide days, both during the wet and dry seasons. The tidal cycle also affected the energy balance closure; we verified a rise in energetic computation during high tides hours, improving the energy balance closure.
